# The Genetic Background of Abnormalities in Metabolic Pathways of Phosphoinositides and Their Linkage with the Myotubular Myopathies, Neurodegenerative Disorders, and Carcinogenesis

**DOI:** 10.3390/biom13101550

**Published:** 2023-10-19

**Authors:** Maria Derkaczew, Piotr Martyniuk, Robert Hofman, Krzysztof Rutkowski, Adam Osowski, Joanna Wojtkiewicz

**Affiliations:** 1 Department of Human Physiology and Pathophysiology, School of Medicine, Collegium Medicum, University of Warmia and Mazury, 10-082 Olsztyn, Poland; 2 Students’ Scientific Club of Pathophysiologists, Department of Human Physiology and Pathophysiology, School of Medicine, University of Warmia and Mazury, 10-082 Olsztyn, Poland; 3 The Nicolaus Copernicus Municipal Polyclinical Hospital in Olsztyn, 10-045 Olsztyn, Poland

**Keywords:** myo-inositol, phosphoinositides, phosphatidylinositol, phosphatidylinositol phosphate, myotubular myopathy, X-linked myotubular myopathy, neurodegenerative disorders, carcinogenesis

## Abstract

Myo-inositol belongs to one of the sugar alcohol groups known as cyclitols. Phosphatidylinositols are one of the derivatives of Myo-inositol, and constitute important mediators in many intracellular processes such as cell growth, cell differentiation, receptor recycling, cytoskeletal organization, and membrane fusion. They also have even more functions that are essential for cell survival. Mutations in genes encoding phosphatidylinositols and their derivatives can lead to many disorders. This review aims to perform an in-depth analysis of these connections. Many authors emphasize the significant influence of phosphatidylinositols and phosphatidylinositols’ phosphates in the pathogenesis of myotubular myopathies, neurodegenerative disorders, carcinogenesis, and other less frequently observed diseases. In our review, we have focused on three of the most often mentioned groups of disorders. Inositols are the topic of many studies, and yet, there are no clear results of successful clinical trials. Analysis of the available literature gives promising results and shows that further research is still needed.

## 1. Introduction

Myo-inositol (MI) is the most common stereoisomer of inositol in eukaryotic cells [1]. MI was discovered by Scherer in 1850, and to this day its properties are still being investigated [2]. The physiological pool of myo-inositol is derived from diet, catabolism of phosphatidylinositols (PIs), phosphatidylinositol phosphates (PIPs)—inositol phosphates (IPs), and form various glucose-included enzymatic reactions [3,4,5]. The main physiological role of myoinositol stands as the precursor of the inositol phospholipids, which after modification by the hormone-stimulated inositol-phospholipid-specific phospholipase C (PLC), generate inositol 1,4,5-trisphosphate (Ins(1,4,5)P3), diacylglycerol (DAG), PI, PIP, IP, glycosylphosphatidylinositols (GPIs), Inositol trisphosphate (IP3), and inositol-phosphoglycans (IPGs) [1,3]. These molecules are used as the ubiquitous second messengers, conveying signals derived by various hormones, e.g., thyroid stimulating hormone (TSH), luteinizing hormone (LH), follicle-stimulating hormone (FSH), and insulin [1,4,6,7]. The interconversions between this group of molecules are conducted by crucial enzymes, whose dysfunction can lead to severe abnormalities, disorders, and illnesses [4,6].

This work aims to present and analyze the documented data concerning the association of cyclitol with pathological processes such as carcinogenesis, myotubular myopathies, and neurodegenerative disorders.

## 2. The Family of Phosphoinositol and Phosphoinositides

Phosphatidylinositol (PtdIns), the starting point of PIP metabolism, is a ubiquitous phospholipid in eukaryotic cells present in various proportions according to the type of membrane. PIPs are all metabolized directly or sequentially from PIs [8]. The structural formulas of phosphoinositol and phosphoinositides are shown in Table 1.

## 3. Routes and Interconversions of PIs

**Figure 1:**
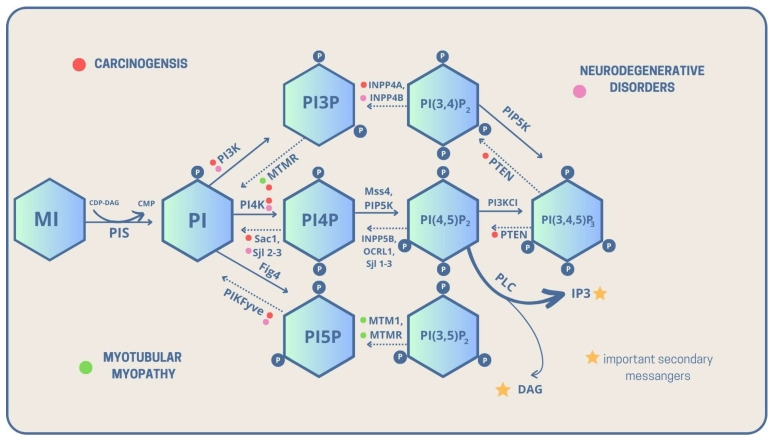
Metabolic interconversions of PIPs with their linkage to carcinogenesis, neurodegenerative diseases, and myotubular myopathies, with highlighted important intracellular secondary messengers [solid lines—phosphorylation, dashed lines—dephosphorylation, CDP-DAG—Cytidine diphosphate diacyloglycerol, CMP—Cytidine monophosphate, DAG—Diacylglycerol, P—phosphate group, PI—Phosphatidylinositol, PI3P—Phosphatidylinositol 3-phosphate, PI4P—Phosphatidylinositol 4-phosphate, PI5P—Phosphatidylinositol 5-phosphate, PI(3,5)P_2_—Phosphatidylinositol 3,5-bisphosphate, PI(3,4)P_2_—Phosphatidylinositol 3,4-bisphosphate, PI(4,5)P_2_—Phosphatidylinositol 4,5-bisphosphate, PI(3,4,5)P_3_—Phosphatidylinositol 3,4,5-trisphosphate, PIS—PI synthase, PLC—Phospholipase C].

As previously mentioned, PI is a key compound and precursor of PIPs, which are all metabolized directly or sequentially from PI [8]. The scheme below presents detailed metabolic routes and interconversions of the PIPs family. The detailed analysis of genes and encoded enzymes is described in the table below Figure 1.

PI itself is a product of the synthesis of cytidine diphosphate diacylglycerol (CPD-DAG) and MI. The reaction is conducted by PI synthase, also called phosphatidylinositol synthase 1 (PIS1) [9,10]. Then, PI phosphorylates are converted into phosphatidylinositol 3-phosphate (PI3P/PtdIns3P) [11]. The conversion is catalyzed by phosphatidylinositol 3 kinase (PI3K) and class III PI 3-kinase—vacuolar protein sorting 34 (Vps34) [12].

In the opposite direction, dephosphorylation occurs, which is conducted by PI3 phosphatases: Yeast myotubularin-related 1 (Ymr1) and Synaptojanin-like proteins 2-3 (Sjl2-3) [13,14]. Next, PI3P is phosphorylated into phosphatidylinositol 3,5-bisphosphate (PI(3,5)P_2_) by PI3P 5-kinase encoded by the Saccharomyces cerevisiae FAB1/PIKfyve genes [15,16,17].

On the other hand, dephosphorylation is conducted by Phosphoinositide 5-phosphatase—FIG4 [18]. Then, PI(3,5)P_2_ can transform into PtdIns5P by dephosphorylation conducted by PI3-phosphatase, MTM1/MTMR1-4, 6-8 [19]. Subsequently, phosphatidylinositol (3,4)-bisphosphate (PI(3,4)P_2_) can turn into PI3P by dephosphorylation catalyzed by PI4-phosphatase: phosphatidylinositol 4,5-bisphosphate 5-phosphatase A and B (INPP4A, INPP4B) [20].

Afterward, PI can also be metabolized into PtdIns4P during phosphorylation conducted by PI4-kinases: Pik1/Stt4 and PI4Kalfa/PI4Kbeta [21,22]. In dephosphorylation, enzymes such as PI4-phosphatases take part: Sjl2-3/Sac1 and SAC1 are similar to the domain of synaptojanin 1 [21,23].

Finally, PtdIns4P can turn into phosphatidylinositol 4,5-bisphosphate PI(4,5)P_2_ during phosphorylation conducted by PI4P 5-kinase: PIP5K α, β, and γ [24]. As for dephosphorylation, it is conducted by PI5-phosphatases: Sjl1-3, INPP5B, and OCRL1 [25,26,27,28].

As previously mentioned in the figure above, every phosphorylation or dephosphorylation reaction is conducted with enzymes such as phosphatidylinositol kinases and phosphatases. Every gene has a specific gene ID. The list of genes, encoded proteins, and their functions are presented in Table 2.

As we can see in the table above, dysfunction of genes or encoded enzymes can lead to various defects and disorders. The range of illnesses varies widely. In this paper, we will focus on *MTM* and *MTMR* gene dysfunctions, neurodegenerative diseases, and carcinogenesis.

## 4. Myotubular Myopathy

Centronuclear myopathy (CNM) is one of the disorders affecting the nervous and muscular systems characterized by two main criteria: symptoms of clinically congenital myopathy and multiple centrally located nuclei in muscle cells confirmed by biopsy [100].

Myotubularin 1 (MTM1) is an enzyme involved in the regulation of phosphoinositides, which are important molecules in cell signaling and membrane trafficking. Mutations in the myotubularin gene *MTM1* which cause human myotubular myopathy dramatically reduce the phosphatase’s ability to dephosphorylate PI3P, affecting the levels of inositol lipid PI3P in myogenesis. In addition, it inhibits the transport of EGFRs to lysosomes, causing the formation of large endosomal vacuoles through the effects of myotubular phosphatase and its interaction with PI(3,5)P_2_. In most patients, mutations in the *MTM1* gene are associated with the recessive X chromosome form (Xq28) [40,41,43,45], while autosomal dominant and recessive forms primarily involve mutations in the dynamin 2 (*DNM2*) gene on chromosome 19p13.2 and the amphisin 2 gene (*BIN1*) on chromosome 2q14 [100].

XLMTM is a specific subtype of myotubular myopathy, which is a rare genetic neuromuscular disorder characterized by severe muscle weakness and hypotonia (low muscle tone) caused by mutations in the *MTM1* gene. As a result, the condition is X-linked, and therefore mainly affects males (2/100,000 male births) [44,45,100]. The symptoms of XLMTM are generally similar to those of other myotubular myopathy subtypes, but they tend to be more severe in males. Newborn males with XLMTM often have profound muscle weakness, leading to severe respiratory difficulties and respiratory failure. The weakened muscles can affect an individual’s ability to breathe, swallow, and move, which makes it essential for affected infants to receive immediate medical attention and respiratory support. Patients with XLMTM can be classified into mild, intermediate, or severe phenotypes based on the amount of ventilation support required. Long-term patients with a severe phenotype are often unable to move and require ventilatory support. Patients with intermediate and mild phenotypes breathe independently for at least a few hours a day and can walk. Moreover, such patients often have coexisting liver disorders [45].

Ross et al. used tissues from human patients and animal models, including dogs with XLMTM, which received increasing doses of recombinant AAV8 vector restoring MTM1 expression (rAAV8-cMTM1). They found that administration of rAAV8-cMTM1 at doses higher than 2.5 × 1013 vg kg^−1^ allowed full recovery of all these cellular defects in dogs with XLMTM [101]. They came to a similar conclusion in their work with an experiment in mice after administration of rAAV8 [102], short-term replacement of myotubularin with a prototypical targeted protein replacement agent (3E10Fv-MTM1) [103], or used the selective inactivation of PI3KC2β kinase activity [104], which improved contractile function and muscle pathology, and thus is associated with a highly promising treatment potential for myotubular myopathy.

*BIN1* modulation as well as *DMN2* reduction may also be an effective treatment strategy for XLCNM, as it restores adequate myofibrillar integrity [105,106].

Normal cell function is regulated by myotubularins belonging to the phosphatidylinositol 3-phosphate phosphatase family PI3P, identified by positional cloning of the *MTM1* gene in patients suffering from X-chromosome-associated myotubular myopathy (with reported specificity toward PI3P) and the myotubularin-related protein 2 (*MTMR2*) gene (hydrolyzes both PI3P and PI(3,5)P_2_ together with myotubularin-related protein 3 (MTMR3) in patients suffering from CMT4B) [42]. Myotubularin-related protein 1 (MTMR1) was shown to use PI3P and/or PI(3,5)P_2_ as substrates. The overall structure was very similar to the previously described structure of MTMR2 [46,47]. It turns out that MTMR2, whose abnormal function is revealed in the neurodegenerative Charcot-Marie-Tooth disease type 4B2, is also highly specific for PI3P as a substrate. In addition, the myotubularin-related phosphatases MTMR1, MTMR3, and myotubularin-related protein 6 (MTMR6) also dephosphorylate PI3P [47]. MTMR3 shows great similarity to myotubularin, primarily due to the catalytic domain, in addition to also having an extension at the C-terminal end that contains the FYVE domain. It can hydrolyze PI3P and PI(3,5)P_2_ and set the pathway for the production of PI5P in the cell. Overexpression of catalytically inactive MTMR3 in cells results in significant formation of vacuolar compartments [48,49].

MTMR3 and myotubularin-related protein 4 (MTMR4) are protein tyrosine phosphatases that dephosphorylate position 3 in PI and generate PI5P from PI(3,5)P_2;_ as well as PI from PI3P. These regulate the production of PI3P, which plays a key role in inhibiting the DNA immune response by regulating the transport of STING, which is an activator of the protein tank-binding kinase (TBK1) that catalyzes the phosphorylation of interferon regulatory factor 3 (IRF3) [51]. Myotubularin-related protein 4 (MTMR4) present in endosomes and regulating their recirculation process has been identified as a novel factor interacting with the ubiquitin ligase neural precursor cell expression protein 4 (Nedd4), which is downregulated during development. *MTMR4* expression decreased in atrophied muscles, while Nedd4 expression increased, and MTMR4 was ubiquitinated by Nedd4, indicating that this new relationship between MTMR4 and Nedd4 may underlie the biological process of muscle degradation [52,53,54]. MTMR8/R9 complex controls a cellular pool of PI3P that has been proposed to be essential in autophagy, a conserved intracellular process for the degradation of cytoplasmic proteins or organelles. Overexpression of both *MTMR8* and *MTMR9* resulted in a significant increase in the level of p62, a protein that is degraded in autophagosomes and is used to monitor autophagy. Mutations in both active and inactive myotubularins (which may play a regulatory role) are associated with diseases such as myotubular myopathy, Charcot-Marie-Tooth (CMT), and others [56,63].

CMT4B is a large group of heterogeneous diseases that are inherited in an autosomal recessive manner and have a progressive sensorimotor neuropathy. The etiopathological basis of MTMR2 and MTMR13, which affect vesicular transport in Schwann cells, is one where the loss of these proteins can lead to uncontrolled myelin folding and, ultimately, to the development of CMT4B disease. Among them, CMT4B is distinguished as having three forms associated with myotubularin family genes: CMT4B1 (*MTMR2* located on chromosome 11q22), CMT4B2 (*MTMR13/SBF2* located on chromosome 11p15) and CMT4B3 (*MTMR5/SBF1*) [107,108].

The disease is childhood-onset and manifests itself mainly through cranial nerve involvement, including glaucoma, vision loss, and other severe disabilities [109].

## 5. Neurodegenerative Diseases

Neurodegenerative diseases are still a significant problem in our aging society [110]. Their occurrence is associated with many factors, such as genetic defects and disturbances in immunological processes. Many neurodegenerative diseases have their cause in the accumulation of extra- and intracellular deposits in the nervous system [111]. Among these disturbances, we can distinguish amyloidoses, tauopathies, α-synucleinopathies, and TDP-43 proteinopathies [110].

Abnormalities in PIP metabolism and their linkage to neurodegenerative disorders has, to date, been mentioned by many authors. Researchers have described the influence of PIPs on the incidence of central nervous system (CNS) diseases through changes in the levels of selected PIPs as well as the enzymes catalyzing their interconversions. The vacuole 14 protein homolog (Vac14) is a frequently mentioned protein, also known as the Associated Regulator of PIKfyve (ArPIKfyve) [112,113].

Zhang et al. postulate that PI(3,5)P_2_ is critical to neuronal health. Vac14 protein is a regulator of the signaling lipid PI(3,5)P_2_ synthesis. The loss of Vac14 results in neurodegeneration processes in the midbrain and peripheral sensory neurons of mice models [114]. Other proteins, such as Fab1/PIKfyve and Fig4/Sac3, also have a regulatory role in the PI(3,5)P_2_ biosynthesis and its relationship with PI5P. Mutations in the genes encoding these proteins lead to the occurrence of neurological diseases, including amyotrophic lateral sclerosis (ALS) and CMT syndrome [115]. For example, Fab1 binds the PI3P and allows it to be converted to PI(3,5)P_2_, and consequently, the appropriate levels of PI(3,5)P_2_ affect the proper functioning of cells in the nervous system. The complex responsible for the Fig1 activity consists of Vac14, Vac7, Fig4, and Atg18 proteins.

Other authors have suggested that changes in the regulation of the phosphatidylinositol-3 kinase (PI3-K) are also linked to many neurodegenerative diseases. One of them is a Nieman-Pick type C disease (NPC), caused by mutations in the *NPC1* and *NPC2* genes. This disorder results in the deposition of neurofibrillary tangles in the CNS, the occurrence of which has been suggested to be associated with increased levels of specific kinases such as phosphatidylinositol 3-kinase (PI3K), glycogen synthase kinase (GSK-3β), and protein kinase B (Akt/PKB). The PI3K cascade leads to the activation of Akt and the inactivation of GSK-3β. Research on the NPC1-deficient mice model has shown major disruptions in the PI3K cascade. The inactivated GSK-3 and phosphorylated Akt were elevated in the neuronal cells, which indicated an unusual level of activity of PI3K in the NPC1-deficient mice brains [116]. Cathepsin D (CD) is one of the key lysosomal proteases. A lack of CD results in a neurodegenerative pediatric disease known as neuronal ceroid lipofuscinosis (NCL/Batten disease). In the research on CD-deficient brains in mice by Walls et al., a decrease in PI3K was observed [117]. 

The phosphatidylinositol 5-phosphate 4-kinases (PI5P4Ks) have been studied for possible therapeutic effects in many diseases, including neurodegenerative ones. PI5P4Ks regulate the cellular level of PI5K and generate a specific pool of PI(4,5)P_2_ products. PI5P4K has free isoforms: α, β, and γ. All of them have different abilities concerning receptor recycling, gene expression regulation, insulin signaling, and cell stress responses [118]. PI5P4Kγ overactivity has a linkage to carcinogenesis and neurodegenerative diseases; thus, it is suggested as a potential therapeutic target. The novel proposed substances in the treatment of neurodegenerative diseases are specific PI5P4Kγ inhibitors and degraders—NIH-12848 and JWZ-1-80 [118,119].

There are also reports with regards to PI(4,5)P_2_ and its connection with neurodegenerative diseases. The immunohistochemical analysis of the neurofibrillary tangles (NFT), characteristic of Alzheimer’s Disease (AD), have proven to be enriched with PI(4,5)P_2_ [120]. The PI(4,5)P_2_ accumulates in neurodegenerative aggregated lipid raft regions. Landman et al. have suggested that PI(4,5)P_2_ imbalance may lead to the pathogenesis of AD, the production of the toxic amyloid β-peptide (Aβ42), and activation of the transient receptor potential melastatin 7 (TRPM7)-associated Mg^2+^-inhibited cation channel (MIC) [121]. In the pathogenesis of AD, Aβ aggregates were found to disrupt many kinases responsible for PI metabolism: membrane-associated phosphatidylinositol-4 kinase (PI4K), PI3K, phosphatidylinositol 4 phosphate kinase (PIP4K), and PI specific phospholipase-C [122].

## 6. Carcinogenesis

Carcinogenesis is a highly complex process involving environmental factors and gene mutations [123]. Critical mutations involve proto-oncogenes, tumor-suppressor genes, and DNA-repair genes. Key principles of cancer are uncontrolled proliferation, metastasis, apoptotic loss, and angiogenesis [124]. Here we present another gene reported to have a contribution to carcinogenesis.

Mutation of myotubularin-related protein 7 gene (*MTMR7*) has been described as contributing to colorectal cancer (CRC) development. MTMR7 itself participates in decreasing insulin-mediated activation of Akt and ERK1/2 signaling, resulting in proliferation reduction of human CRC cells. In human colorectal cancers, MTMR7 has been down-regulated, which has been related to a poor prognosis [62].

PI3K mis-activation has been widely reported in cancer diseases [69,125,126]. Its activation can be signaled via various pathways including mTOR, JAK2/STAT5, Akt, or RTK [69], which gives a promising location for the development of target drugs, but can also be responsible for therapy resistance. Phosphatidylinositol 3-kinase catalytic subunit type 3 (PIK3C3), a subunit of the PI3K complex, takes part in the formation, initiation, and maturation of autophagosomes [127]. It has been shown that, in the condition of oncogenic herpesvirus KSHV infection, the expression of PIK3C3 is upregulated, and takes part in tumor progression and metastasis [68]. It is worth mentioning that various drugs have been developed to interfere with the PI3K/AKT/mTOR axis. So far, pan and isoform-specific PI3K inhibitors have been developed, and some have had promising results [128,129,130]. The PI3K inhibitor copanlisib has been clinically approved for follicular lymphoma, and idelalisib for chronic lymphocytic leukemia, follicular lymphoma, and small lymphocytic lymphoma [128,131]. There is also data suggesting the usage of copanlisib against solid tumors and other non-Hodgkin lymphomas may be warranted [129,132].

The PKR/PI4K2A axis, which takes part in the clearance of misfolded proteins in lysosomes, has been revealed to be a potential drug target—inhibiting tumor growth in the lung and breast [74].

The phosphatase and tensin homolog (PTEN) is a commonly known cancer suppressor that acts mainly via inhibiting PI3K/Akt activation [133]. This molecule controls the cell cycle, driving apoptosis among pathological conditions [134]. Mutations of *PTEN* are commonly known for the link to the PTEN hamartoma tumor syndrome (PHTS), which is characterized by a greater risk of cancer occurrence, includes Cowden syndrome (CS), Bannayan-Riley-Ruvalcaba syndrome (BRRS), and PTEN-related Proteus syndrome (PS) [135,136]. To date, there has been some effort put into establishing PTEN as a potential drug target. For example, the research of inducing PTEN de-ubiquitination has resulted in the cessation of its degradation, which subsequently has driven PTEN activity in inhibiting the AKT signaling pathway and tumor growth [137,138]. Other members of the PTEN family, TPTE and PTEN homologous inositol lipid phosphatase (TPIP), reported to occur in at least three forms (alpha, beta, and gamma), have been reported to act as tumor suppressors, as the overexpression of this molecule leads to cell proliferation inhibition and apoptosis induction [99,139].

Upregulation of Inositol polyphosphate 4-phosphatase type II (INPP4B) leads to colon cancer cell proliferation, growth, and survival, which is associated with increased activation of PI3K/Akt and SGK3. Of note, in this study, it turned out that PTEN inhibited INPP4B-mediated PI3K signaling activation [36]. This finding is consistent with the study on pancreatic cancer using mice models, where INPP4B had additionally been found to inhibit apoptosis. In addition, INPP4B expression was correlated with a positive resection margin, poor overall survival, and disease-free survival. Moreover, INPP4B could be a potential diagnostic marker of use almost equal to or greater than that of other established diagnostic markers, such as CA125, AFP, and CEA [140].

Mutation of *SACM1L*, a gene for phosphatidylinositol-3-phosphatase SAC1, is common among cancers [95]. In the breast cancer model, the loss of SAC1 function has led to their increased migration and invasion. Interestingly, the loss of SAC1 corresponded with increased levels of Golgi PI4P [141].

A protein and lipid kinase FYVE finger-containing phosphoinositide kinase (PIKfyve) has been described as taking a crucial role in regulating the endosomal system and transport in the cells, as well as to participate in downstream membrane signals, remodeling the cytoskeleton and even innate immune responses [142,143]. Mutation in gene *PIKFYVE* for this kinase has been investigated as having a potential role in carcinogenesis, and there is also some evidence that this gene can be an oncogene [144]. This makes PIKfyve a potential treatment target. Studies on PIKfyve inhibitors showed promising results in reducing multiple myeloma and non-Hodgkin lymphoma cell viability via inducing cell vacuolization, rupture of the plasma membrane, and non-apoptotic death [86,145,146].

## 7. Conclusions

Summing up, MI is a forerunner of many derivatives, including PIs, PIPs, IPs, GPIs, and IPGs, which take part in transmitting various signals in the eukaryotic cells. That is why gene mutations for these molecules can play a pivotal role in the development of many widespread diseases, such as cancers or neurodegenerative disorders. Cancer diseases and neurodegenerative disorders, such as AD, are wide-spread problems among developing countries, and the number of patients has been increasing for many years.

Consequently, there is a growing need for studies to better explain the development of these diseases. In our review, we focused on phosphatydylinositols and the linkage between their gene mutations and connected disorders. These alterations contribute to, e.g., carcinogenesis, enabling cancer cells to get their key principles, such as uncontrolled proliferation, apoptosis inhibition, and increased migration. Other diseases whose pathogenesis is related to the improper functioning of PIPs are myotubular myopathies. Their impaired function of PIPs disrupts myogenesis, cellular compartmental organization, and the accumulation of improper metabolites, leading to macroscopic muscle defects.

In neurodegenerative disorders, it has been described that mutations of genes for PIPs lead to disruption of intracellular trafficking and, once again, the accumulation of defective metabolites. Subsequently, it disturbs the very complex and active functioning of the neural cells. All of the studies conducted on PIP gene mutations and mentioned in our review give a better insight into many common diseases. The connections described make PIPs and their genes potential drug targets and diagnostic markers in the disorders mentioned above.

Our review provides an expanded overview of the most important links between PIP mutations and the occurrence of diseases. To obtain more information, further studies focused on the particular mutations are needed.

## Figures and Tables

**Table 1:** Structural formulas of phosphoinositol and phosphoinositides.

PI Phosphatidylinositol	** 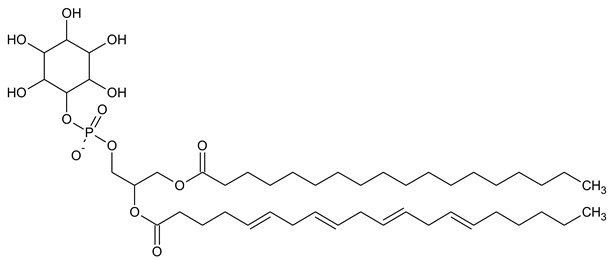 **
PI3P Phosphatidylinositol 3-phosphate	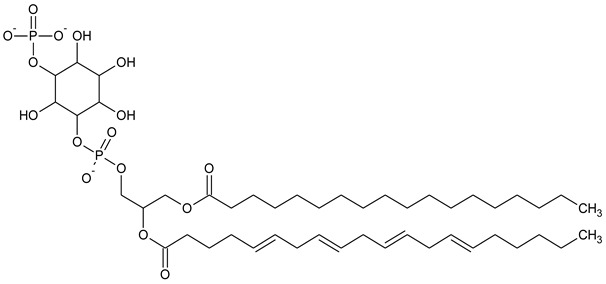
PI(3,5)P_2_ Phosphatidylinositol 3,5-bisphosphate	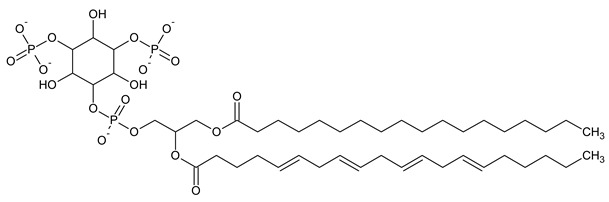
PI4P Phosphatidylinositol 5-phosphate	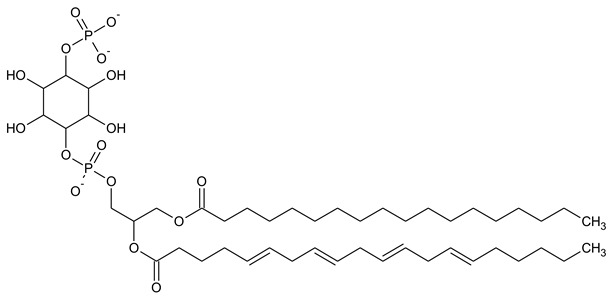
PI(3,4)P_2_ Phosphatidylinositol 3,4-bisphosphate	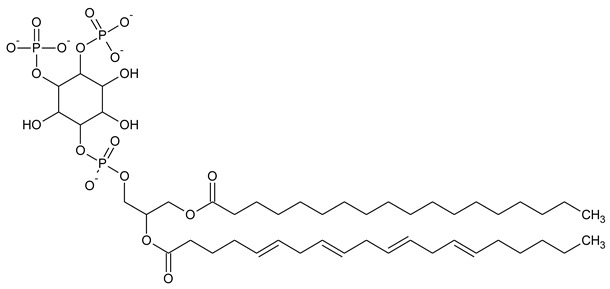
PI(3,4,5)P_3_ Phosphatidylinositol 3,4,5-trisphosphate	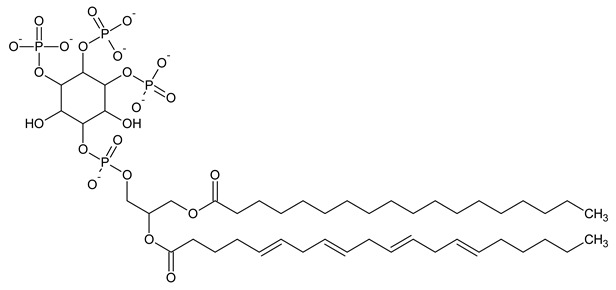
PI5P Phosphatidylinositol 5-phosphate	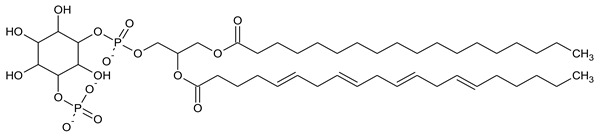
PI(4,5)P_2_ Phosphatidylinositol 4,5-bisphosphate	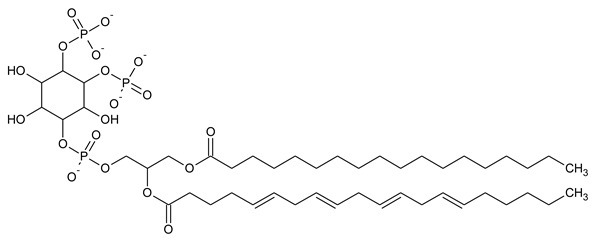

**Table 2:** List of genes and proteins with their functions.

Gene	Protein	Gene ID	Protein Function	Protein Function Literature	Associated Illness or Defect	Associated Illness or Defect Literature
*INPP4A*	Inositol polyphosphate-4-phosphatase type I A	3631	- catalyzes the hydrolysis of the 4-position phosphate of PI(3,4)P2, inositol 1,3,4-triphosphate and inositol 1,4-bisphosphate - antagonizes the Phosphatidylinositol-3 kinase (PI3K)-protein kinase B (PKB, Akt) signaling pathway by dephosphorylating phosphoinositides - modulates cell cycle progression and cell survival - may protect neurons from excitotoxic cell death by regulating the synaptic localization of cell surface N-methyl-D-aspartate-type glutamate receptors (NMDARs) and NMDAR-mediated excitatory postsynaptic current - protects neurons from excitotoxic cell death and maintains the functional integrity of the brain	Junko Sasaki et al., 2010 [29] Ivan Ivetac et al., 2005 [30] Rituparna Chaudhuri et al., 2018 [31]	- alacrimia - achalasia - mental retardation - temporal lobe epilepsy	Junko Sasaki et al., 2010 [29] Li Wang et al., 2018 [32]
*INPP4B*	Inositol polyphosphate 4-phosphatase type II	8821	- catalyzes the hydrolysis of the 4-position phosphate of PI(3,4)P_2_, inositol 1,3,4-trisphosphate and inositol 3,4-trisphosphate - takes part in the late stages of macropinocytosis by dephosphorylating PI(3,4)P_2_ in membrane ruffles - the lipid phosphatase activity is crucial for tumor suppressor function - it antagonizes the PI3K-Akt/PKB signaling pathway by dephosphorylating phosphoinositides and modulating cell cycle progression and cell survival	Sandra M. Lopez et al., 2013 [33] Masashi Maekawa et al., 2014 [34] Christina Gewinner et al., 2009 [35]	- cancers: colon, thyroid, melanoma, prostate, and breast cancer	ST Guo et al., 2015 [36] Shuyu Zhai et al., 2019 [37]
*INPP5B*	Type II inositol 1,4,5-trisphosphate 5-phosphatase	3633	- hydrolyzes PI(4,5)P_2_ and PI(1,4,5)P_3_ and modulates cellular signaling events	Jefferson et al., 1995 [38]	- Lowe syndrome	Susan P. Bothwell et al., 2010 [39]
*MTM1*	Myotubularin 1	4534	- dephosphorylates PI3P and PI(3,5)P_2_ - dephosphorylates phosphotyrosine- and phosphoserine-containing peptides - dephosphorylates phosphotyrosine- and phosphoserine-containing peptides - negatively regulates epidermal growth factor receptor (EGFR) degradation by regulation of EGFR trafficking from the late endosome to the lysosome - plays a role in vacuolar formation and morphology - regulates desmin intermediate filament assembly and architecture - plays a role in mitochondrial morphology and positioning - required for skeletal muscle maintenance - stabilizes myotubularin-related protein 12 (MTMR12) protein levels in skeletal muscles	Blondeau et al., 2000 [40] Taylor et al., 2000 [41] Schaletzky et al., 2003 [42] Tsujita et al., 2004 [43] Gupta et al., 2013 [44]	- X-linked myotubular myopathy (XLMTM)	M’elanie Annoussamy et al., 2021 [45]
*MTMR1*	Myotubularin-related protein 1	8776	- has high specificity to PI3P	Soo-A Kim et al., 2002 [46] Seoung Min Bong et al., 2016 [47]	- XLMTM - Charcot-Marie-Tooth disease type 4B (CMT4B)	Soo-A Kim et al., 2001 [46]
*MTMR2*	Myotubularin-related protein 2	8898	- has phosphatase activity towards PI3P and PI(3,5)P_2_	Soo-A Kim et al., 2002 [46]	- XLMTM - CMT4B	Soo-A Kim et al., 2002 [46]
*MTMR3*	Myotubularin-related protein 3	8897	- has phosphatase activity towards PI3P and PI(3,5)P_2_	Donna M. Walker, 2001 [48] Runxiang Zhao, 2001 [49]	- XLMTM - CMT4B	Amit Lahiri et al., 2015 [50]
*MTMR4*	Myotubularin-related protein 4	9110	- dephosphorylates proteins phosphorylated on serine (Ser), threonine (Thr), and tyrosine (Tyr) residues - phosphorylates PIP3 - dephosphorylates proteins phosphorylated on Ser, Thr, and Tyr residues	Dyaningtyas Dewi Pamungkas Putri et al., 2019 [51] Pamela J. Plant et al., 2009 [52] Kumar et al., 2017 [53]	- Mulibrey nanism syndrome - XLMTM - inflammatory bowel disease	Monica J. Naughtin et al., 2010 [54]
*MTMR6*	Myotubularin-related protein 6	9107	- dephosphorylates PI3P and PI(3,5)P_2_ - binds with high affinity to PI(3,5)P_2_ - negatively regulates the proliferation of reactivated CD4+ T-cells - myotubularin-related protein 6 (MTMR6) in complex with myotubularin-related protein 9 (MTMR9) negatively regulates DNA damage-induced apoptosis - the formation of the MTMR6-MTMR9 complex stabilizes both MTMR6 and MTMR9 protein levels - MTMR6 takes part in the late stages of macropinocytosis by dephosphorylating PI3P in membrane ruffles - MTMR6 acts as a negative regulator of Potassium Calcium-Activated Channel Subfamily N Member 4 (KCNN4)/calcium-activated potassium channel (KCa3.1) channel activity in CD4+ T-cells by decreasing intracellular levels of PI3P	Jun Zou et al., 2009 [55] Jun Zou et al., 2012 [56] Masashi Maekawa et al., 2014 [34] Shekhar Srivastava et al., 2005 [57]	- XLMTM	Yasuhiro Mochizuki et al., 2013 [58]
*MTMR7*	Myotubularin-related protein 7	9108	- takes part in dephosphorylation of PI3P and Ins(1,3)P_2_	Philip Weidner et al., 2016 [59]	- colorectal cancer - Creutzfeldt-Jakob disease	Dan Zhao et al., 2019 [60] Philip Weidner et al., 2016 [59] Pascual Sanchez-Juan et al., 2012 [61] Philip Weidner et al., 2020 [62]
*MTMR8*	Myotubularin-related protein 8	55613	- has phosphatase activity towards PI3P and PI(3,5)P_2_ - MTMR8 in complex with MTMR9 negatively regulates autophagy	Jun Zou et al., 2012 [56] Ki-Young Yoo et al., 2015 [63]	- XLMTM - galactosemia	Jun Zou et al., 2012 [56]
*OCRL-1*	OCRL inositol polyphosphate-5-phosphatase	4952	- has PI(4,5)P_2_ 5-phosphatase activity	Pascale Gaudet et al., 2011 [64]	- Lowe syndrome - Dent disease type 2	Maria Antonietta De Matteis et al., 2017 [65]
*PIK3C3*	Phosphatidylinositol 3-kinase catalytic subunit type 3	5289	- is a catalytic subunit of the PI3K complex that mediates the formation of PtdIns3P - takes part in multiple membrane trafficking pathways: PI3KC3-C1 is involved in the initiation of autophagosomes and PI3KC3-C2 in the maturation of autophagosomes and endocytosis - promotes endoplasmic reticulum membrane curvature formation prior to vesicle budding - is involved in the transport of lysosomal enzyme precursors to lysosomes	Bao-cun Zhang et al., 2020 [66] Sigrid B. Thoresen et al., 2010 [67]	- Kaposi Sarcoma - XLMTM with excessive autophagy	Haidai Hu et al., 2015 [68]
*PI3K*	Phosphatidylinositol 3-kinase	39089293	- PI3K is a central enzyme in a signaling pathway - mediates cellular responses to insulin and other growth factors and mediates insulin-dependent regulation of glucose metabolism	David A. Fruman et al., 2017 [69]	- insulin resistance - diabetes mellitus - carcinogenesis	David A. Fruman et al., 2014 [70] Alexandre Arcaro et al., 2007 [71]
*PI4K2A*	Phosphatidylinositol 4-kinase type 2 alpha	55361	- phosphatidylinositol 4-Kinase Type 2 Alpha (PI4K2A) plays a crucial role in endocytosis, Golgi function, protein sorting, and membrane trafficking - membrane-bound PI4-kinase catalyzes the phosphorylation of PI	Emma L. Clayton et al., 2013 [72] Qiangjun Zhou et al., 2014 [73]	- congenital aphakia - Hermansky-Pudlak syndrome - carcinogenesis	Apar Pataer et al., 2020 [74]
*PI4KA*	Phosphatidylinositol 4-kinase alpha	5297	- acts on PI in the early stage of the production of the second messenger inositol-1,4,5,-trisphosphate	T Gehrmann et al., 1999 [75] Fubito Nakatsu et al., 2012 [76]	- perisylvian polymicrogyria - cerebellar hypoplasia - heparin cofactor II deficiency	Alistair T. Pagnamenta et al., 2015 [77]
*PIKFYVE*	1-phosphatidylinositol 3-phosphate 5-kinase	200576	- maintenance of endomembrane homeostasis - endocytic-vacuolar pathway - lysosomal trafficking - nuclear transport - stress- or hormone-induced signaling and cell cycle progression - the PI(3,5)P2 regulatory complex regulates synthesis and turnover of PI(3,5)P_2_ - catalyzes the phosphorylation of PtdIns3P on the fifth hydroxyl of the inositol ring to form PI(3,5)P_2_ - catalyzes the phosphorylation of PI on the fifth hydroxyl of the inositol ring to form PtdIns5P - has serine-protein kinase activity and can autophosphorylate and transphosphorylate - takes part in crucial endosome operations such as fission and fusion in the course of endosomal cargo transport - is required for the maturation of early into late endosomes, phagosomes, and lysosomes - regulates vacuole maturation and nutrient recovery following engulfment of macromolecules - initiates the redistribution of accumulated lysosomal contents back into the endosome network - is a fundamental regulator of the morphology, degradative activity, and protein turnover of the endolysosomal system in macrophages and platelets - generates reactive oxygen species (ROS) in neutrophils, which is crucial to perform chemotaxis and undertake phagosome fusion with lysosomes - is crucial in the processing and presentation of antigens by major histocompatibility complex class II (MHC class II) mediated by cathepsin S (CTSS) - regulates melanosome biogenesis by controlling the delivery of proteins from the endosomal compartment to the melanosome - is essential for systemic glucose homeostasis, mediates insulin-induced signals for endosome and actin remodeling in the course of glucose transporter type 4 (GLUT4) translocation and glucose uptake activation - supports microtubule-based endosome-to-Golgi network cargo transport, through association with Sperm-Associated Antigen 9 (SPAG9) and rab9 effector protein with Kelch motifs (RABEPK) - mediates EGFR trafficking to the nucleus	Shisheva et al., 2012 [78] Kim et al., 2007 [79] Sbrissa et al., 2007 [80] Sbrissa et al., 2012 [81] Krishna et al., 2016 [82] Dayam et al., 2017 [83] Liggins et al., 2018 [84] Baranov et al., 2019 [85]	- Huntington’s disease - fleck and corneal dystrophy - multiple myeloma	Cecilia Bonolo de Campos et al., 2020 [86]
*PTEN*	Phosphatidylinositol 3,4,5-trisphosphate 3-phosphatase and dual-specificity protein phosphatase PTEN	5728	- acts as a dual-specificity protein phosphatase, dephosphorylating tyrosine-, serine-, and threonine-phosphorylated proteins. - in addition, it acts as a lipid phosphatase, removing the phosphate in the D3 position of the inositol ring from PI(3,4,5)P_3_, PI(3,4)P_2_, PtdIns3P and inositol 1,3,4,5-tetrakisphosphate - It is also a negative regulator of PI3K chemotaxis pathways	Iijima et al., 2002 [87] Funamoto et al., 2002 [88] Wessels et al., 2007 [89] McMains et al., 2008 [90] Gruver et al., 2008 [91] Maeda et al., 2008 [92]	- PTEN hamartoma tumor syndrome	Yehia and Eng, 2021 [93]
*SAC1*	Phosphatidylinositol-3-phosphatase SAC1	22908	- it is a phosphoinositide phosphatase which catalyzes the hydrolysis of PI(3,5)P2 - it can catalyze the hydrolysis of PI3P and PI4P - it is required for normal cell morphogenesis, cell wall synthesis, and actin organization	Zhong et al., 2005 [94]	- Alzheimer’s disease - polycystic kidney disease - various forms of cancer	Del Bel and Brill, 2018 [95]
*STT4*	Phosphatidylinositol 4-kinase STT4	851014	- it acts on PI at the early stage in the production of the second messenger inositol 1,4,5-trisphosphate. - STT4 has crucial functions in the protein kinase C 1 (PKC1) pathway	Ghaemmaghami et al., 2003 [96]	- improper functioning of PKC1 protein kinase pathway	Ghaemmaghami et al., 2003 [96]
*SYNJ1*	Synaptojanin-1	8867	- the pit is a phosphatase that acts on various phosphoinositides, including PI4P, PI(4,5)P_2,_ and PI(3,4,5)P_3_ - it takes part in clathrin-mediated endocytosis - it hydrolyzes PIP2 bound to actin regulatory proteins resulting in the rearrangement of actin filaments downstream of tyrosine kinase and ASH/GRB2	Hardies et al., 2016 [97]	- Parkinson Disease - epileptic encephalopathy	Quadri et al., 2013 [98]
*TPIP α*	TPTE and PTEN homologous inositol lipid phosphatase	93492	- it takes part in regulating phosphoinositide signaling on the endoplasmic reticulum - represents a tumor suppressor and functional homolog of PTEN in some tissues	Walker et al., 2001 [99]	- carcinogenesis	Walker et al., 2001 [99]

## Data Availability

Not applicable.

## Abbreviations

3E10Fv-MTM1	Prototypical targeted protein replacement agent
AD	Alzheimer’s disease
ALS	Amyotrophic lateral sclerosis
ArPIKfyve	Associated regulator of PIKfyve
BRRS	Bannayan-Riley-Ruvalcaba syndrome
BIN1	Bridging Integrator 1
CD	Cathepsin D
CDP-DAG	Cytidine diphosphate diacylglycerol
CMP	Cytidine monophosphate diacylglycerol
CMT	Charcot-Marie-Tooth disease
CMT4B	Charcot-Marie-Tooth disease type 4B
CNM	Centronuclear myopathy
CNS	Central nervous system
CS	Cowden syndrome
CTSS	Cathepsin S
Cvt	Cytoplasm to vacuole transport
DAG	Diacylglycerol
DNM2	dynamin 2
EGF	epidermal growth factor
EGFR	Epidermal growth factor receptor
FSH	Follicle stimulating hormone
GLUT 4	Glucose transporter type 4
GPI	Glycosyl-phosphatidylinositol
GSK-3β	Glycogen synthase kinase
INPP4A	Phosphatidylinositol 4,5-bisphosphate 5-phosphatase A
INPP4B	Phosphatidylinositol 4,5-bisphosphate 5-phosphatase B
Ins(1,4,5)P3	Inositol 1,4,5-trisphosphate
IP	Inositol Phosphate
IP3	Inositol trisphosphate
IPG	Inositol-phosphoglycan
IRF3	Interferon regulatory transcription factor 3
JNK	c-Jun N-terminal kinase
KCa3.1	Calcium-activated potassium channel
KCNN4	Potassium calcium-activated channel subfamily N member 4
LH	Luteinizing hormone
MHC class II	Major histocompatibility complex class II
MI	Myo-inositol
MTM	Myotubular myopathy
MTM1	Myotubularin 1
MTMR1	Myotubularin-related protein 1
MTMR2	Myotubularin-related protein 2
MTMR3	Myotubularin-related protein 3
MTMR4	Myotubularin-related protein 4
MTMR6	Myotubularin-related protein 6
MTMR7	Myotubularin-related protein 7
MTMR8	Myotubularin-related protein 8
MTMR9	Myotubularin-related protein 9
MTMR12	Myotubularin-related protein 12
NCL	Neuronal ceroid lipofuscinosis
Nedd4	Neural precursor cell expression protein 4
NFT	Neurofibrillary tangles
NMDAR	N-methyl-D-aspartate-type glutamate receptor
NPC	Nieman-Pick type C
NPC 1	Nieman-Pick type C gene 1
NPC 2	Nieman-Pick type C gene 2
P	Phosphate group
PHTS	PTEN hamartoma tumor syndrome
PI(3,4)P_2_/PtdIns(3,4)P_2_	Phosphatidylinositol 3,4-bisphosphate
PI(3,4,5)P_3_/PtdIns(3,4,5)P_3_	Phosphatidylinositol 3,4,5-trisphosphate
PI(3,5)P_2_/PtdIns(3,5)P_2_	Phosphatidylinositol 3,5-bisphosphate
PI(4,5)P_2_ /PtdIns(4,5)P_2_/PIP2	Phosphatidylinositol 4,5-bisphosphate
PI/PtdIns	Phosphatidylinositol
PI3K	Phosphatidylinositol-3 kinase
PI3P	Phosphatidylinositol 3-phosphate
PI3P/PtdIns3P	Phosphatidylinositol 3-phosphate
PI4K	Phosphatidylinositol-4 kinase
PI4K2A	Phosphatidylinositol 4-Kinase Type 2 Alpha
PI4P/PtdIns4P	Phosphatidylinositol 4-phosphate
PI5P	Phosphatidylinositol 5-phosphate
PI5P/PtdIns5P	Phosphatidylinositol 5-phosphate
PI5P4K	Phosphatidylinositol 5-phosphate 4-kinase
PLC	Phospholipase C
PIP/PtdInsP	Phosphatidylinositol phosphate
PIP4K	Phosphatidylinositol 4 phosphate kinase
PIS1	Phosphatidylinositol synthase 1
PKB/Akt	Protein kinase B
PKC	Protein kinase C
PS	Proteus syndrome
rAAV8-cMTM1	recombinant AAV8 vector restoring MTM1 expression
RABEPK	Rab9 effector protein with Kelch motifs
ROS	Reactive oxygene species
Ser	Serine
Sjl2-3	Synaptojanin-like proteins 2-3
SPAG9	Sperm Associated Antigen 9
TBK 1	tank-binding kinase 1
Thr	hreonine
TSH	Thyroid stimulating hormone
Tyr	Tyrosine
Vac 14	Vacuole 14 protein homolog
XLMTM	X-linked myotubular myopathy
Ymr1	Yeast myotubularin related 1
